# Task-based assessment of neck CT protocols using patient-mimicking phantoms—effects of protocol parameters on dose and diagnostic performance

**DOI:** 10.1007/s00330-020-07374-8

**Published:** 2020-11-05

**Authors:** Paul Jahnke, Juliane Conzelmann, Ulrich Genske, Maximilian Nunninger, Michael Scheel, Bernd Hamm, Torsten Diekhoff

**Affiliations:** 1 Department of Radiology, Charité – Universitätsmedizin Berlin, corporate member of Freie Universität Berlin, Humboldt-Universität zu Berlin, and Berlin Institute of Health, Charitéplatz 1, Berlin, 10117 Germany; 2 Berlin Institute of Health (BIH) , Anna-Louisa-Karsch-Str. 2, Berlin, 10178 Germany; 3 Department of Neuroradiology, Charité – Universitätsmedizin Berlin, corporate member of Freie Universität Berlin, Humboldt-Universität zu Berlin, and Berlin Institute of Health, Charitéplatz 1, Berlin, 10117 Germany

**Keywords:** Tomography, X-ray computed, Phantoms, imaging, Health physics, Neck, Radiation protection

## Abstract

**Objectives:**

To assess how modifying multiple protocol parameters affects the dose and diagnostic performance of a neck CT protocol using patient-mimicking phantoms and task-based methods.

**Methods:**

Six patient-mimicking neck phantoms containing hypodense lesions of 1 cm diameter and 30 HU contrast and one non-lesion phantom were examined with 36 CT protocols. All possible combinations of the following parameters were investigated: 100- and 120-kVp tube voltage; tube current modulation (TCM) noise levels of SD 7.5, 10, and 14; pitches of 0.637, 0.813, and 1.388; filtered back projection (FBP); and iterative reconstruction (AIDR 3D). Dose-length products (DLPs) and lesion detectability (assessed by 14 radiologists) were compared with the clinical standard protocol (120 kVp, TCM SD 7.5, 0.813 pitch, AIDR 3D).

**Results:**

The DLP of the standard protocol was 25 mGy•cm; the area under the curve (AUC) was 0.839 (95%CI: 0.790–0.888). Combined effects of tube voltage reduction to 100 kVp and TCM noise level increase to SD 10 optimized protocol performance by improving dose (7.3 mGy•cm) and detectability (AUC 0.884, 95%CI: 0.844–0.924). Diagnostic performance was significantly affected by the TCM noise level at 120 kVp (AUC 0.821 at TCM SD 7.5 vs. 0.776 at TCM SD 14, *p* = 0.003), but not at 100-kVp tube voltage (AUC 0.839 at TCM SD 7.5 vs. 0.819 at TCM SD 14, *p* = 0.354), the reconstruction method at 100 kVp (AUC 0.854 for AIDR 3D vs. 0.806 for FBP, *p* < 0.001), but not at 120-kVp tube voltage (AUC 0.795 for AIDR 3D vs. 0.793 for FBP, *p* = 0.822), and the tube voltage for AIDR 3D reconstruction (*p* < 0.001), but not for FBP (*p* = 0.226).

**Conclusions:**

Combined effects of 100-kVp tube voltage, TCM noise level of SD 10, a pitch of 0.813, and AIDR 3D resulted in an optimal neck protocol in terms of dose and diagnostic performance. Protocol parameters were subject to complex interactions, which created opportunities for protocol improvement.

**Key Points:**

*• A task-based approach using patient-mimicking phantoms was employed to optimize a CT system for neck imaging through systematic testing of protocol parameters.*

*• Combined effects of 100-kVp tube voltage, TCM noise level of SD 10, a pitch of 0.813, and AIDR 3D reconstruction resulted in an optimal protocol in terms of dose and diagnostic performance.*

*• Interactions of protocol parameters affect diagnostic performance and should be considered when optimizing CT techniques.*

**Electronic supplementary material:**

The online version of this article (10.1007/s00330-020-07374-8) contains supplementary material, which is available to authorized users.

## Introduction

Computed tomography (CT) acquisition protocols should provide diagnostic image quality at the lowest possible dose in line with the ALARA principle (as low as reasonably achievable). Protocol settings used in clinical routine determine diagnostic reliability and dose exposure of patients [1]. On a larger scale, they affect the overall radiation burden to the population from CT examinations [2]. Previous work has shown that CT acquisition protocols and related dose exposure vary substantially across facilities [3, 4]. Protocol optimization thus offers significant potential for improving patient safety.

Prior efforts to optimize protocols harmonized dose exposure across CT facilities through protocol revision by expert panels and best practice sharing [5, 6]. Such programs were shown to be valuable in reducing dose and identifying dose outliers. However, they were not intended to assess diagnostic image quality, which means that they could neither exclude unacceptable image quality nor identify optimal protocols that strike the best balance between dose and image quality. Protocol optimization for such purposes can be defined in terms of determining how to better use a system’s imaging capabilities for obtaining adequate diagnostic information at the lowest possible dose.

Today, CT protocols routinely involve technologies that reduce dose, but also have complex effects on image properties. Especially the use of iterative reconstruction algorithms is associated with complex interactions between noise, texture, contrast, and spatial resolution [7–9], which are not adequately assessed with traditional metrics such as contrast-to-noise ratios [10]. In light of this situation, methods are of interest that reliably predict clinical image performance and allow comparison of protocols independently of the CT techniques involved. The recent report of the AAPM task group 233 on CT performance evaluation has highlighted the role of task-based methods for such purposes [11].

Task-based methods assess image quality by testing how well CT images enable an observer to perform detection tasks that are similar to clinical diagnostic tasks of radiologists [12]. The simplest of such tasks is the detection of a signal (e.g., a low-contrast lesion) against a uniform phantom background [13]. However, patients are not uniform, and the complexity of background texture affects image properties and detection outcomes [7, 14]. A further development towards assessing CT protocols more realistically would therefore involve quantitative assessment of detection outcomes in anatomically more realistic phantoms.

Recent work has introduced 3D-printed phantoms that realistically mimic a patient’s contrast medium–enhanced neck and contain low-contrast lesions for task-based image quality assessment [15]. The present study builds on these methods and uses patient-mimicking phantoms with embedded low-contrast lesions to assess protocol parameters in neck CT imaging. The hypothesis was that, through the testing of protocol parameters in a realistic setting, optimized parameter combinations that make better use of the imaging techniques available on a specific CT system can be found to ensure diagnostic image quality at lower dose for a given application. Based on these assumptions, the aim was to assess how modifying multiple protocol parameters affects the dose and diagnostic performance of a neck CT protocol using patient-mimicking phantoms and task-based methods.

## Methods

### Study design

The institutional ethics committee approved the study and waived informed consent. Ethics approval was obtained to perform the study with seven 3D printed phantoms mimicking a patient’s neck (six phantoms containing a low-contrast lesion, one non-lesion phantom). The phantoms were examined with 36 different CT protocols. Image quality was assessed by 14 radiologists using task-based methods (8064 readings in total). The protocols were analyzed for dose and image quality and compared with our clinical standard protocol.

### Phantoms

**Fig. 1:**
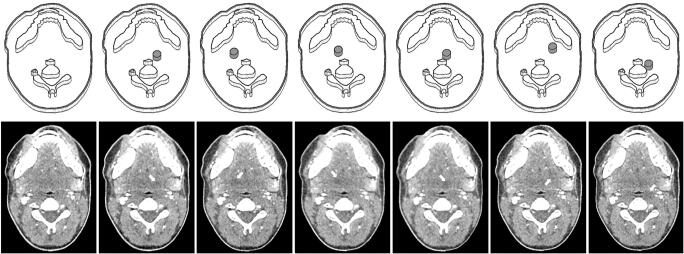
Drawings and CT images of the phantoms used for protocol assessment. Lesions are drawn in gray and indicated by white arrows in the CT images. The CT images shown here were acquired with 120-kVp tube voltage, TCM SD of 7.5, and a pitch of 0.813 and reconstructed with AIDR 3D (corresponding to the reference protocol used in this study). All images are displayed with window level 40 and window width 350

Seven anatomically identical phantoms mimicking a patient’s neck were created based on previously published methods [15]. Briefly, the phantoms were produced from seven different versions of a CT image of a patient’s neck: the original, unmodified CT image and six versions of the same image, where lesions of 1 cm diameter were inserted through pixelwise subtraction of 30 HU in a circular region of interest in different locations throughout the parapharyngeal space. Radiopaque 3D printing with potassium-iodide-doped ink and paper-based 3D printing were used to produce the phantoms with 1 cm thickness [16, 17]. The method of phantom creation was the same as described in more detail previously [15], except that all lesions had 30 HU contrast and were distributed throughout the parapharyngeal space. This lesion contrast was selected to create lesions at the interface between detectable and undetectable based on that previous study. Six phantoms thus contained one low-contrast lesion of 1 cm diameter and 30 HU contrast in different locations of the parapharyngeal space. One phantom did not contain any lesion. Figure 1 shows illustrations and CT images of the phantoms. For image acquisition, the 1-cm-thick phantoms were inserted into a full-size head and neck phantom as shown in suppl. fig. 1.

### CT acquisition

**Fig. 2:**
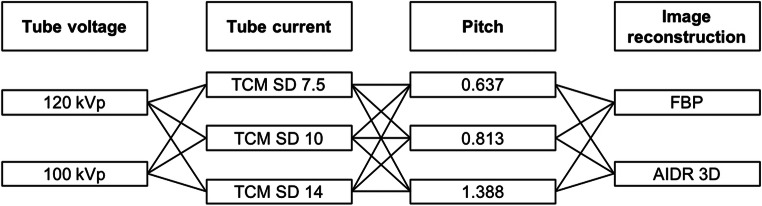
Acquisition flow chart. Two tube voltages, three tube currents, three pitch factors, and two image reconstruction algorithms were combined, resulting in a total of 36 possible combinations, which were investigated

Images were acquired on a Canon Aquilion Prime CT scanner (Canon Medical Systems). All acquisitions covered 4 cm in z-direction, with the inserted phantoms in the center along the z-axis. The phantoms were imaged with a total of 36 different acquisition protocols to investigate all possible combinations of different tube voltage, tube current, pitch, and reconstruction settings (Fig. 2). For all acquisitions, 100-kVp tube voltage corresponded to the recommended setting of the automatic tube potential selection system. The tube current modulation (TCM) noise levels corresponded to the CT settings for high quality (SD 7.5), quality (SD 10), and standard (SD 14) as recommended by the CT vendor. Two acquisitions per protocol and phantom were performed. Images were reconstructed with 0.5-mm slice thickness and a soft tissue kernel (FC08). For analysis, the current clinical standard neck protocol was used as reference: 120-kVp, TCM SD of 7.5, 0.813 pitch, and AIDR 3D.

### Dose assessment

Dose-length products (DLPs) were assessed. To account for the short scan length of 4 cm, the contribution of overscanning to the DLP was calculated using previously published methods [18] and reduced to 20%. The contribution of overscanning to the DLP was thus made equivalent to a scan coverage of 20 cm in z-direction.

### Image quality assessment

Fourteen radiologists with prior training in neck CT imaging participated in the readings. Their experience ranged from 2 to 15 years (mean: 4.9, median: 4). Readers were presented with 16 images per acquisition protocol: one image per acquisition of the lesion phantoms and two images per acquisition of the non-lesion phantom. Two images of the non-lesion phantom were included to adjust the proportion between images showing lesions (for which six phantoms were used) and images not showing lesions (for which only one phantom was available). Each reader was thus presented with a total of 576 images (6 lesion phantoms × 2 acquisitions × 1 image per acquisition × 36 protocols + 1 non-lesion phantom × 2 acquisitions × 2 images per acquisition × 36 protocols). For each presented image, readers were asked to indicate whether a lesion was present. If a lesion was deemed present, they were asked to draw a region of interest containing the entire lesion. Readers were blinded to the experimental design in that they were unaware of how many different lesion positions were possible in the study setting. All readings were performed on diagnostic monitors (Eizo RadiForce RX250, Eizo Corporation) using in-house developed software.

### Data analysis

The intersection over union (IOU) between the lesion ground truth and reader selection was calculated [19]. Reader responses were classified into the following: (1) no lesion marked, (2) lesion marked on negative samples, (3) IOU = 0, (4) IOU ≤ 0.5, and (5) IOU > 0.5. Receiver operating characteristic statistics were performed, and the area under the curve (AUC) was determined per reader and acquisition protocol. Based on these results, interrater reliability was determined using the intraclass correlation coefficient. AUC differences between each protocol and the reference protocol along with one-sided 95% confidence intervals (CI) were calculated. A non-inferiority analysis was performed to compare the AUC between each protocol and the reference protocol [20]. The limit of non-inferiority was set before the analysis and considered at 5% of the mean AUC value of the reference protocol (mean AUC 0.839, non-inferiority limit −0.042). Non-inferiority was assumed when the lower limit of the 95% CI (one-sided) was greater than the limit of non-inferiority. Superiority was assumed when the lower limit of the 95% CI (one-sided) was greater than 0. Inferiority was assumed when the upper limit of the 95% CI (one-sided) was less than 0. In addition, the effects of tube voltage, tube current, pitch, and image reconstruction parameters on AUC results were analyzed using a general linear model; *p* values for multiple comparisons were adjusted with Sidak’s method. Differences were interpreted as significant when *p* < 0.05.

## Results

### Dose and detectability results

**Fig. 3:**
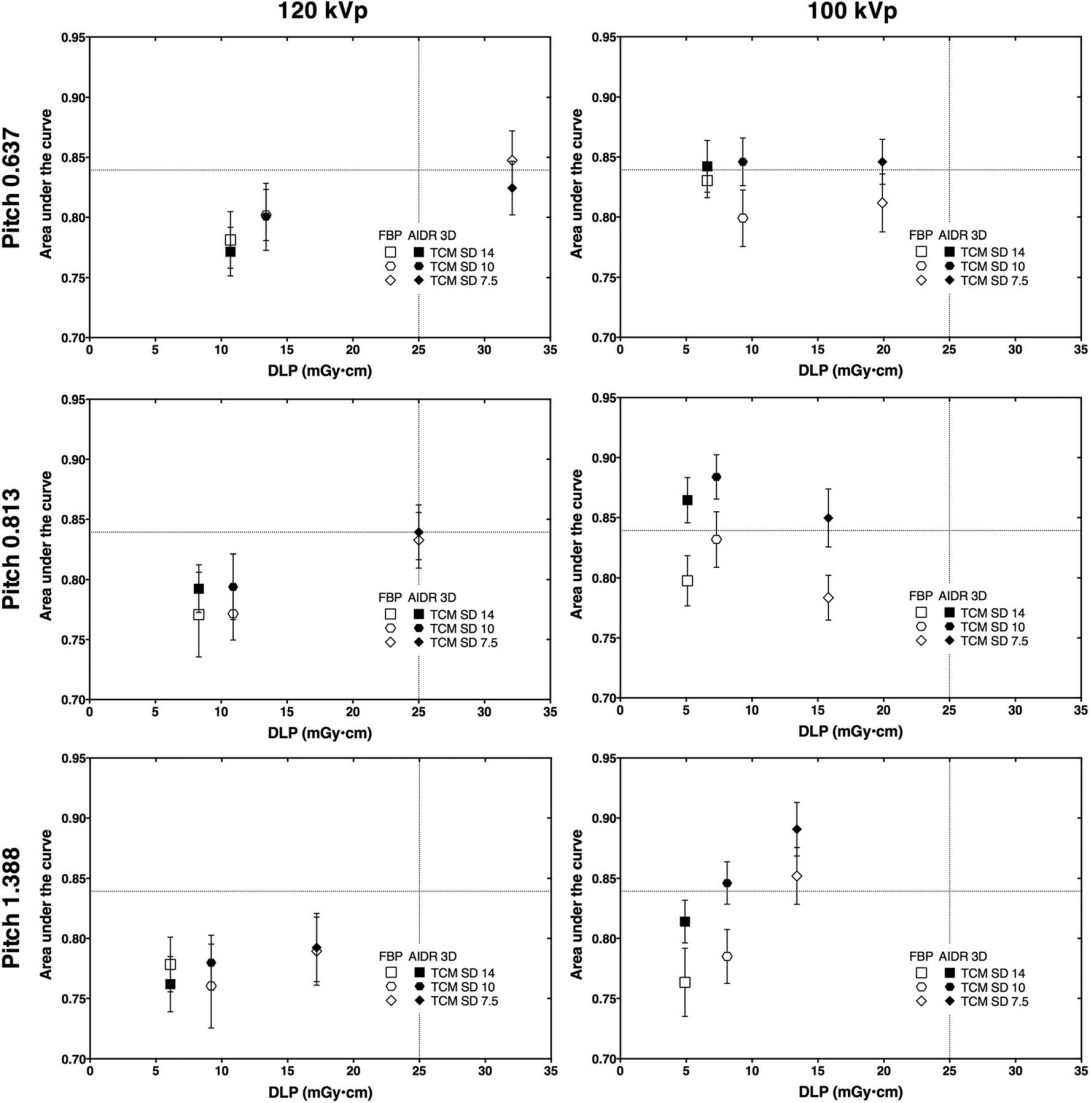
Dose and detectability results. Error bars indicate standard errors of the mean. Grid lines indicate the reference protocol (120-kVp tube voltage, TCM SD of 7.5, pitch of 0.813, AIDR 3D)

The intraclass correlation coefficient between AUC results of the 14 participating radiologists was 0.73 (95% CI: 0.58 to 0.84). Reading time for all 576 images was approximately 90 min per participant. The reference protocol had a DLP of 25 mGy•cm with a mean AUC across all readers of 0.839 (95% CI: 0.790 to 0.888). Figure 3 shows the dose and detectability results for all protocols in relation to the reference protocol. Except for a pitch reduction to 0.637, all protocol modifications resulted in a lower dose. Suppl. Table 1 summarizes dose and detectability results, and Suppl. figure 2 provides a series of exemplary CT images acquired with the 36 protocols investigated.

### Protocol improvement

**Fig. 4:**
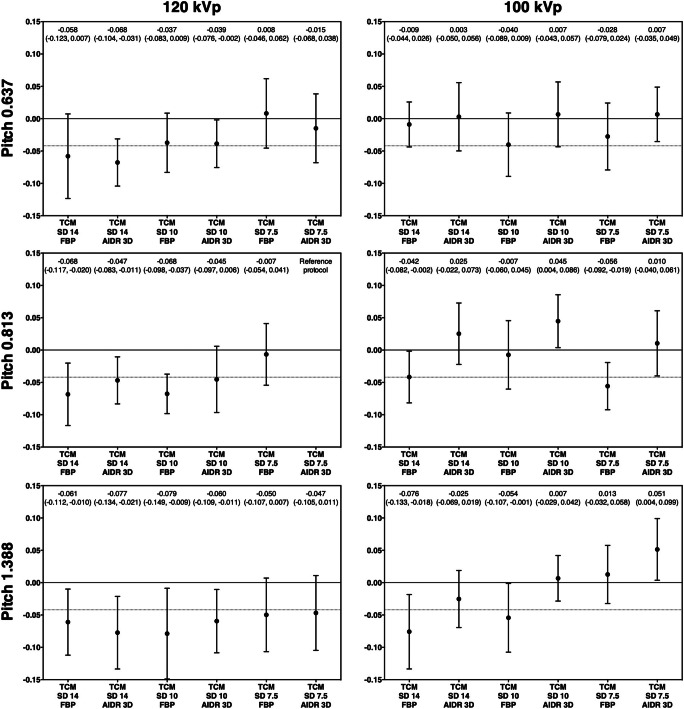
Results of the non-inferiority analysis. The non-inferiority limit (indicated by grid lines) was set to −0.042, corresponding to 5% of the mean AUC value of the reference protocol (120-kVp tube voltage, TCM SD of 7.5, pitch of 0.813, AIDR 3D). Mean AUC differences between each protocol and the reference protocol along with one-sided 95% confidence intervals are indicated on top

**Fig. 5:**
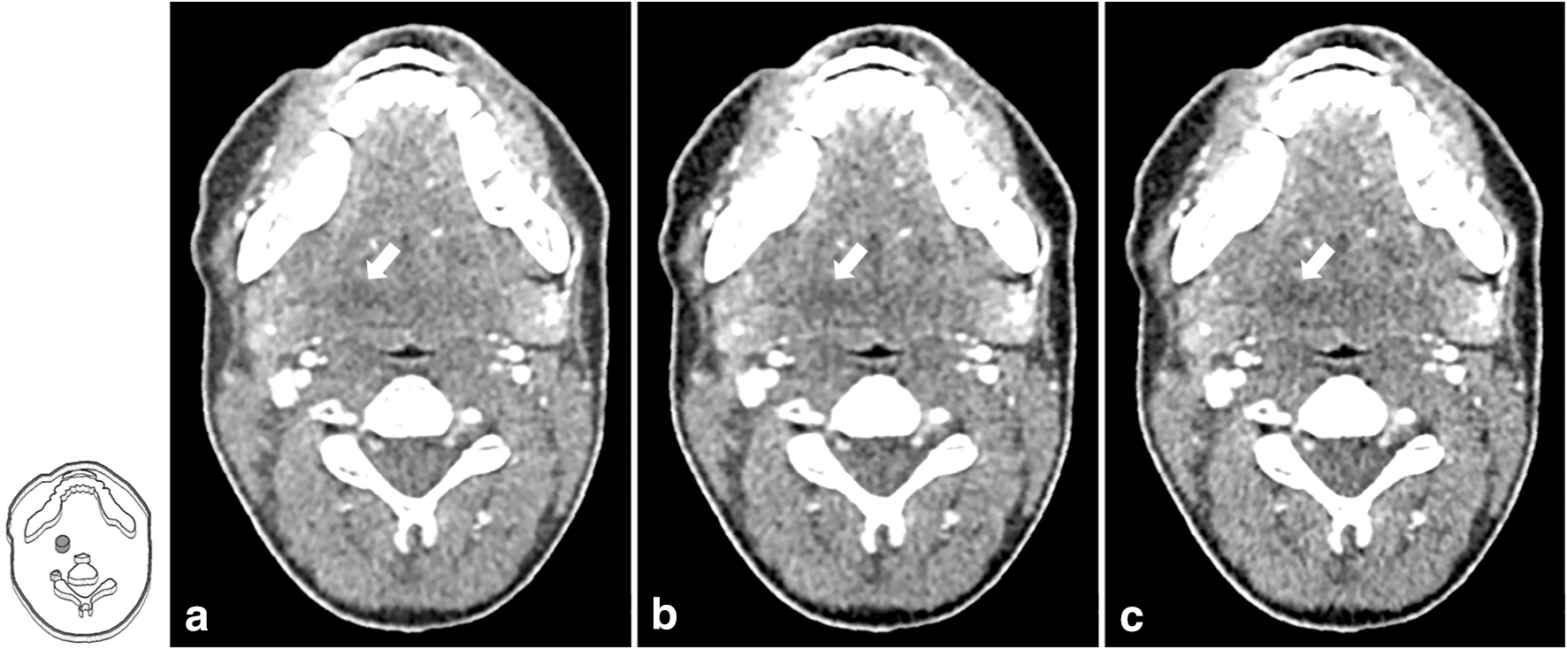
Comparison of CT images acquired with the reference protocol and two protocols that yielded superior detectability results. **a** Reference protocol (120 kVp, TCM SD of 7.5, pitch of 0.813, AIDR 3D). **b** Reduced tube voltage and tube current (100 kVp, TCM SD of 10, pitch of 0.813, AIDR 3D). **c** Reduced tube voltage and increased pitch (100 kVp, TCM of 7.5, pitch of 1.388, AIDR 3D). The drawing indicates the lesion position, and lesions are additionally indicated by white arrows in the CT images. Images are displayed with window level/window width 40/350 at 120 kVp and 80/350 at 100 kVp

Figure 4 presents the results of the non-inferiority analysis. Compared with the reference protocol, seven protocols yielded non-inferior detectability at a reduced dose and thus improved protocol performance (Table 1). The strongest dose reduction at non-inferior detectability was achieved by reducing tube voltage to 100 kVp and increasing the TCM noise level to SD 14 (AUC 0.865, 95% CI: 0.824 to 0.905; DLP 5.1 mGy•cm). Two protocols reduced dose and yielded superior detection results, which means that dose exposure and image quality were improved simultaneously. One of these protocols used a lower tube voltage of 100 kVp and a higher pitch of 1.388, which increased the AUC to 0.891 (95% CI: 0.842 to 0.939) and reduced the DLP to 13.4 mGy•cm. The other protocol used a lower tube voltage of 100 kVp and higher TCM noise level of 10, which increased the AUC to 0.884 (95% CI: 0.844 to 0.924) and reduced the DLP even further to 7.3 mGy•cm. For comparison, Fig. 5 presents CT images acquired with these two protocols and the reference protocol.

**Table 1:** Comparison of the reference protocol with seven improved protocols that reduced dose and achieved non-inferior or superior diagnostic performance

Tube voltage (kVp)	Tube current	Pitch	Image reconstruction	DLP (mGy•cm)	Mean AUC (95% confidence intervals)	Non-inferiority or superiority
120	TCM SD 7.5	0.813	AIDR 3D	25.0	0.839 (0.790 to 0.888)	Reference protocol
100	TCM SD 7.5	0.637	AIDR 3D	19.9	0.846 (0.806 to 0.887)	Non-inferiority shown
100	TCM SD 7.5	0.813	AIDR 3D	15.8	0.850 (0.798 to 0.902)	Non-inferiority shown
100	TCM SD 10	0.813	AIDR 3D	7.3	0.884 (0.844 to 0.924)	Superiority shown
100	TCM SD 14	0.813	AIDR 3D	5.1	0.865 (0.824 to 0.905)	Non-inferiority shown
100	TCM SD 7.5	1.388	FBP	13.4	0.852 (0.801 to 0.903)	Non-inferiority shown
100	TCM SD 7.5	1.388	AIDR 3D	13.4	0.891 (0.842 to 0.939)	Superiority shown
100	TCM SD 10	1.388	AIDR 3D	8.1	0.846 (0.808 to 0.884)	Non-inferiority shown

### Protocol parameter effects

**Fig. 6:**
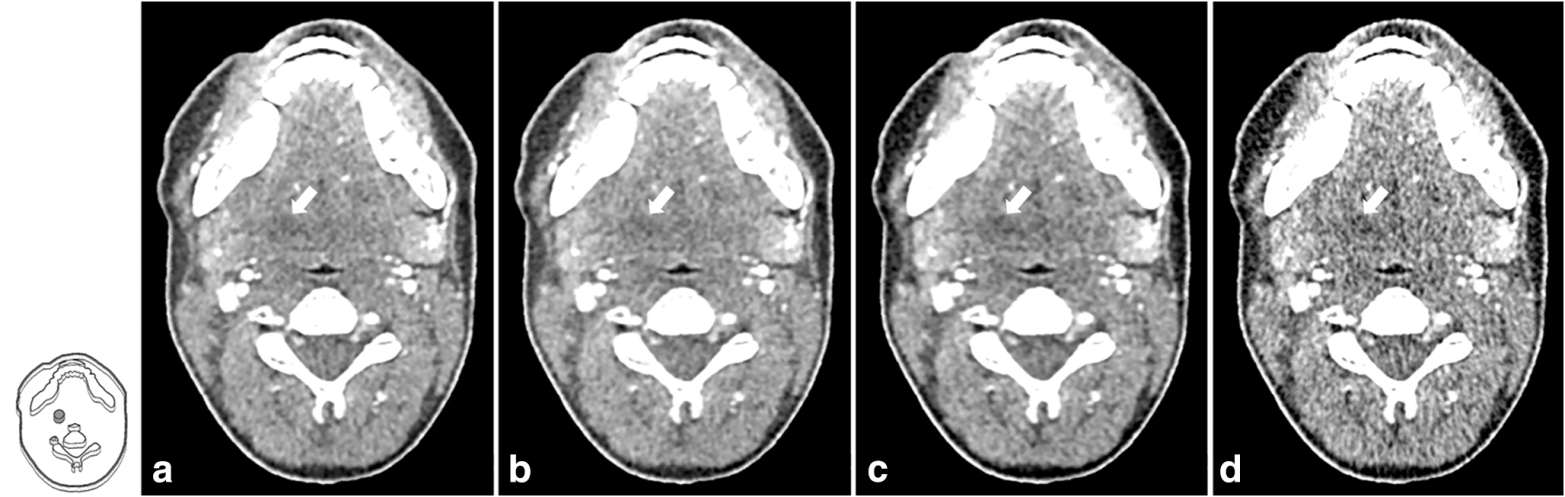
CT images illustrating the effects of tube voltage reduction, tube current reduction, and the image reconstruction method used. **a** Reference protocol (120 kVp, TCM SD of 7.5, pitch of 0.813, AIDR 3D). **b** Reduced tube current (120 kVp, TCM SD of 14, pitch of 0.813, AIDR 3D). **c** Reduced tube voltage and tube current (100 kVp, TCM SD of 14, pitch of 0.813, AIDR 3D). **d** Same acquisition parameters as **c** but reconstructed with FBP instead of AIDR 3D. The drawing indicates the lesion position, and lesions are additionally indicated by white arrows in the CT images. Images are displayed with window level/window width 40/350 at 120 kVp and 80/350 at 100 kVp

Figure 6 shows a comparison between a CT image acquired with the reference protocol and three CT images illustrating the effects of different parameter combinations: (1) acquired with reduced tube current (TCM SD 14) and reconstructed with AIDR 3D, (2) acquired with reduced tube voltage (100 kVp) and reduced tube current (TCM SD 14) and reconstructed with AIDR 3D, and (3) the same acquisition parameters as in (2) but reconstructed with FBP. A higher TCM noise level resulted in inferior detectability when combined with 120-kVp tube voltage while detectability was unchanged when combined with a reduced tube voltage of 100 kVp. Likewise, image reconstruction with FBP at reduced tube voltage and higher TCM noise level resulted in inferior detectability while reconstruction with AIDR 3D did not (Table 2).

**Table 2:** Summarized results show combined effects of tube voltage, tube current, and image reconstruction method in comparison with the reference protocol

Tube voltage (kVp)	Tube current	Pitch	Image reconstruction	DLP (mGy•cm)	Mean AUC (95% confidence intervals)	Inferiority or non-inferiority
120	TCM SD 7.5	0.813	AIDR 3D	25.0	0.839 (0.790 to 0.888)	Reference protocol
120	TCM SD 14	0.813	AIDR 3D	8.3	0.792 (0.750 to 0.835)	Inferiority shown
100	TCM SD 14	0.813	AIDR 3D	5.1	0.865 (0.824 to 0.905)	Non-inferiority shown
100	TCM SD 14	0.813	FBP	5.1	0.798 (0.752 to 0.843)	Inferiority shown

These results were consistent with the results obtained with other parameter combinations. In a comparison across all protocols, a TCM noise level increase from 7.5 to 14 reduced detectability at 120-kVp tube voltage (AUC 0.821, 95% CI: 0.802 to 0.840 vs. 0.776, 95% CI: 0.757 to 0.795; *p* = 0.003). However, at 100 kVp, detectability was less markedly degraded by a TCM noise level increase from 7.5 to 14 (AUC 0.839, 95% CI: 0.820 to 0.858 vs. 0.819, 95% CI: 0.800 to 0.837; *p* = 0.354) and decreased only significantly in conjunction with a pitch of 1.388 (AUC 0.871, 95% CI: 0.839 to 0.904 vs. 0.789, 95% CI: 0.756 to 0.821; *p* = 0.001). Compared with FBP, AIDR 3D improved detectability at 100 kVp (AUC 0.806, 95% CI: 0.791 to 0.821 vs. 0.854, 95% CI: 0.838 to 0.869; *p* < 0.001), but not at 120-kVp tube voltage (AUC 0.793, 95% CI: 0.777 to 0.808 vs. 0.795, 95% CI: 0.780 to 0.810; *p* = 0.822). Conversely, lowering the tube voltage from 120 to 100 kVp significantly improved detectability with the use of AIDR 3D (*p* < 0.001), but not with FBP for image reconstruction (*p* = 0.226). These findings are summarized in Tables 3 and 4.

**Table 3:** Comparison of high and low tube voltage and tube current protocols. Mean area under the curve values and 95% confidence intervals across all protocols and readers are shown

	TCM SD of 7.5	TCM SD of 14	
120 kVp	0.821 (0.802 to 0.840)	0.776 (0.757 to 0.795)	*p* = 0.003
100 kVp	0.839 (0.820 to 0.858)	0.819 (0.800 to 0.837)	*p* = 0.354
	*p* = 0.184	*p* = 0.002

**Table 4:** Comparison of protocols using high and low tube voltages and FBP and AIDR 3D for image reconstruction. Mean area under the curve values and 95% confidence intervals across all protocols and readers are shown

	FBP	AIDR 3D	
120 kVp	0.793 (0.777 to 0.808)	0.795 (0.780 to 0.810)	*p* = 0.822
100 kVp	0.806 (0.791 to 0.821)	0.854 (0.838 to 0.869)	*p* < 0.001
	*p* = 0.226	*p* < 0.001	

## Discussion

CT protocol optimization has significant potential for improving patient safety by reducing dose exposure and/or enhancing the diagnostic yield of CT images. Optimizing protocols for clinical care requires the use of methods that are predictive of clinical performance. To this end, the present study assessed neck CT protocols using patient-mimicking phantoms and task-based methods. Thirty-six protocols were evaluated and compared with a clinical protocol combining 120-kVp tube voltage, a TCM noise level of 7.5, a pitch of 0.813, and image reconstruction with AIDR 3D. Protocol parameters were varied and their effect on dose exposure and detectability of low-contrast lesions in the parapharyngeal space was analyzed.

We identified seven protocols that reduced dose without yielding inferior detection results compared with the clinical reference protocol. The strongest dose reduction at non-inferior detectability was achieved with 100-kVp tube voltage, a TCM SD of 14, a pitch of 0.813, and AIDR 3D (DLP reduction from 25 to 5.1 mGy•cm). Two protocols achieved superior detectability, which means that diagnostic performance was improved while dose was reduced. Based on these results, an optimal protocol can be derived, which uses 100-kVp tube voltage, a TCM SD of 10, a pitch of 0.813, and AIDR 3D for image reconstruction. This protocol improves detectability while reducing the DLP from 25 to 7.3 mGy•cm compared with the reference protocol.

Each of the scan parameters varied in the present study individually affects dose and image quality. Lower tube voltages reduce dose while increasing noise and contrast [21] and have been reported previously to maintain or improve low-contrast detectability [22, 23]. Higher TCM noise levels reduce dose and increase noise and have been found to degrade low-contrast detectability in previous studies [24, 25]. Iterative reconstruction is noise- and contrast-dependent and affects noise, texture, and spatial resolution [8, 9]. Low-contrast detectability has been previously reported to improve with IR or to be equivalent to FBP [26, 27].

The results of the present study reflect how different combinations of these effects jointly affect a detection task in a clinical setting. For example, higher lesion contrast compensated for higher noise in most protocols with 100-kVp tube voltage, so that tube current could be reduced without compromising detectability. AIDR 3D reconstruction, which is noise- and contrast-dependent, enabled positive effects of tube voltage reduction on detectability, supporting previous reports of better lesion detection on 100-kVp IR than on 120-kVp FBP images [28]. Conversely, in our experiments, advantages of AIDR 3D over FBP were also more significant when a lower tube voltage (corresponding to greater noise and contrast) was used. The results illustrate the complexity that arises from varying multiple parameters and that conclusions regarding the advantages and disadvantages of particular CT techniques should consider the protocol context in which they were studied.

This complexity makes it desirable to compare different protocol scenarios directly and systematically and to predict their performance in clinical practice. The experimental study presented here therefore used task-based methods that have been developed for such purposes. However, our approach differed from most previous studies in that anatomical and not uniform phantoms were used, which is of relevance because texture and anatomic detail have been shown to affect image properties [7], human lesion perception [29], and the relationship between dose and image quality [30]. We therefore consider the search tasks used in the present study to be more complex and realistic, which should make the results more representative of clinical practice. A focus of future work will be to further develop and adapt the methodology for application to other anatomical regions and diagnostic tasks. Another focus will be to investigate whether the phantom design can be modified to facilitate image acquisition, e.g., by integrating multiple lesions simultaneously.

Limitations of the present study include that the results only apply to contrast medium–enhanced neck imaging and the CT scanner and techniques used in this study. For example, IR algorithms from different vendors have been shown to have different effects on low-contrast detectability [31, 32]. Also, due to the small number of phantoms investigated, signal locations were not completely random. However, the risk of bias was reduced as readers were unaware of the number of phantoms and signal locations. Detectability was assessed by human observers, which is most representative of the performance of radiologists in the clinical setting but also subject to significant variability and time-consuming. Future work could address this limitation by using a model observer approach [33].

CT protocols vary considerably between scanners and institutions and it is likely that many patients could be examined more efficiently. Our study presents an approach to test and optimize protocol parameters in a realistic context in order to use the imaging techniques of a CT system more efficiently to deliver diagnostic information. The results illustrate how interactions between protocol parameters affect diagnostic performance, which should be borne in mind when assessing the diagnostic effects of CT techniques.

## Electronic supplementary material

ESM 1 (DOCX 1.32 mb)

## Abbreviations

AIDR 3D: Adaptive iterative dose reduction 3D
AUC: Area under the curve
CI: Confidence interval
DLP: Dose-length product
FBP: Filtered back projection
HU: Hounsfield unit
IOU: Intersection over union
IR: Iterative reconstruction
SD: Standard deviation
TCM: Tube current modulation
