# Supplementary material for: Task-based assessment of neck CT protocols using patient-mimicking phantoms—effects of protocol parameters on dose and diagnostic performance
Source: Eur Radiol. 2020 Nov 5;31(5):3177–86. doi: 10.1007/s00330-020-07374-8 (PMC8043932; doi:10.1007/s00330-020-07374-8)

Suppl. table 1: Summarized acquisition parameters, computed tomography dose index (CTDI), dose-length products (DLPs), area under the curve (AUC) values, and results of the non-inferiority analysis for all protocols.

| **Tube voltage** | **Tube current** | **Pitch** | **Image reconstruction** | **CTDI (mGy)** | **DLP (mGy•cm)** | **Mean AUC (95% confidence intervals)** | **Non-inferiority** | **Superiority** | **Inferiority** |
| --- | --- | --- | --- | --- | --- | --- | --- | --- | --- |
| 120 kVp | TCM SD 7.5 | 0.637 | FBP | 7.2 | 32.1 | 0.848 (0.794 to 0.901) | not shown | not shown | not shown |
| 120 kVp | TCM SD 7.5 | 0.637 | AIDR 3D | 7.2 | 32.1 | 0.824 (0.776 to 0.873) | not shown | not shown | not shown |
| 120 kVp | TCM SD 10 | 0.637 | FBP | 3 | 13.4 | 0.802 (0.756 to 0.848) | not shown | not shown | not shown |
| 120 kVp | TCM SD 10 | 0.637 | AIDR 3D | 3 | 13.4 | 0.801 (0.741 to 0.861) | not shown | not shown | shown |
| 120 kVp | TCM SD 14 | 0.637 | FBP | 2.4 | 10.7 | 0.781 (0.731 to 0.832) | not shown | not shown | not shown |
| 120 kVp | TCM SD 14 | 0.637 | AIDR 3D | 2.4 | 10.7 | 0.772 (0.728 to 0.815) | not shown | not shown | shown |
| 120 kVp | TCM SD 7.5 | 0.813 | FBP | 5.6 | 25.0 | 0.833 (0.783 to 0.882) | not shown | not shown | not shown |
| 120 kVp | TCM SD 7.5 | 0.813 | AIDR 3D | 5.6 | 25.0 | 0.839 (0.790 to 0.888) | Reference | Reference | Reference |
| 120 kVp | TCM SD 10 | 0.813 | FBP | 2.5 | 10.9 | 0.772 (0.724 to 0.819) | not shown | not shown | shown |
| 120 kVp | TCM SD 10 | 0.813 | AIDR 3D | 2.5 | 10.9 | 0.794 (0.735 to 0.853) | not shown | not shown | not shown |
| 120 kVp | TCM SD 14 | 0.813 | FBP | 1.9 | 8.3 | 0.771 (0.695 to 0.847) | not shown | not shown | shown |
| 120 kVp | TCM SD 14 | 0.813 | AIDR 3D | 1.9 | 8.3 | 0.792 (0.750 to 0.835) | not shown | not shown | shown |
| 120 kVp | TCM SD 7.5 | 1.388 | FBP | 3.8 | 17.2 | 0.789 (0.728 to 0.851) | not shown | not shown | not shown |
| 120 kVp | TCM SD 7.5 | 1.388 | AIDR 3D | 3.8 | 17.2 | 0.792 (0.731 to 0.854) | not shown | not shown | not shown |
| 120 kVp | TCM SD 10 | 1.388 | FBP | 2.2 | 9.2 | 0.760 (0.685 to 0.836) | not shown | not shown | shown |
| 120 kVp | TCM SD 10 | 1.388 | AIDR 3D | 2.2 | 9.2 | 0.780 (0.730 to 0.829) | not shown | not shown | shown |
| 120 kVp | TCM SD 14 | 1.388 | FBP | 1.4 | 6.1 | 0.778 (0.729 to 0.828) | not shown | not shown | shown |
| 120 kVp | TCM SD 14 | 1.388 | AIDR 3D | 1.4 | 6.1 | 0.762 (0.712 to 0.812) | not shown | not shown | shown |
| 100 kVp | TCM SD 7.5 | 0.637 | FBP | 4.5 | 19.9 | 0.812 (0.760 to 0.864) | not shown | not shown | not shown |
| 100 kVp | TCM SD 7.5 | 0.637 | AIDR 3D | 4.5 | 19.9 | 0.846 (0.806 to 0.887) | shown | not shown | not shown |
| 100 kVp | TCM SD 10 | 0.637 | FBP | 2.2 | 9.3 | 0.799 (0.749 to 0.850) | not shown | not shown | not shown |
| 100 kVp | TCM SD 10 | 0.637 | AIDR 3D | 2.2 | 9.3 | 0.846 (0.803 to 0.889) | not shown | not shown | not shown |
| 100 kVp | TCM SD 14 | 0.637 | FBP | 1.5 | 6.6 | 0.830 (0.800 to 0.861) | not shown | not shown | not shown |
| 100 kVp | TCM SD 14 | 0.637 | AIDR 3D | 1.5 | 6.6 | 0.842 (0.796 to 0.889) | not shown | not shown | not shown |
| 100 kVp | TCM SD 7.5 | 0.813 | FBP | 3.7 | 15.8 | 0.784 (0.743 to 0.824) | not shown | not shown | shown |
| 100 kVp | TCM SD 7.5 | 0.813 | AIDR 3D | 3.7 | 15.8 | 0.850 (0.798 to 0.902) | shown | not shown | not shown |
| 100 kVp | TCM SD 10 | 0.813 | FBP | 1.8 | 7.3 | 0.832 (0.782 to 0.882) | not shown | not shown | not shown |
| 100 kVp | TCM SD 10 | 0.813 | AIDR 3D | 1.8 | 7.3 | 0.884 (0.844 to 0.924) | shown | shown | not shown |
| 100 kVp | TCM SD 14 | 0.813 | FBP | 1.2 | 5.1 | 0.798 (0.752 to 0.843) | not shown | not shown | shown |
| 100 kVp | TCM SD 14 | 0.813 | AIDR 3D | 1.2 | 5.1 | 0.865 (0.824 to 0.905) | shown | not shown | not shown |
| 100 kVp | TCM SD 7.5 | 1.388 | FBP | 3.1 | 13.4 | 0.852 (0.801 to 0.903) | shown | not shown | not shown |
| 100 kVp | TCM SD 7.5 | 1.388 | AIDR 3D | 3.1 | 13.4 | 0.891 (0.842 to 0.939) | shown | shown | not shown |
| 100 kVp | TCM SD 10 | 1.388 | FBP | 1.8 | 8.1 | 0.785 (0.737 to 0.833) | not shown | not shown | shown |
| 100 kVp | TCM SD 10 | 1.388 | AIDR 3D | 1.8 | 8.1 | 0.846 (0.808 to 0.884) | shown | not shown | not shown |
| 100 kVp | TCM SD 14 | 1.388 | FBP | 1.2 | 4.9 | 0.763 (0.702 to 0.825) | not shown | not shown | shown |
| 100 kVp | TCM SD 14 | 1.388 | AIDR 3D | 1.2 | 4.9 | 0.814 (0.776 to 0.853) | not shown | not shown | not shown |

Suppl. fig. 1: Photographs illustrating how a 1 cm thick phantom is inserted into a full-size head and neck phantom for image acquisition.


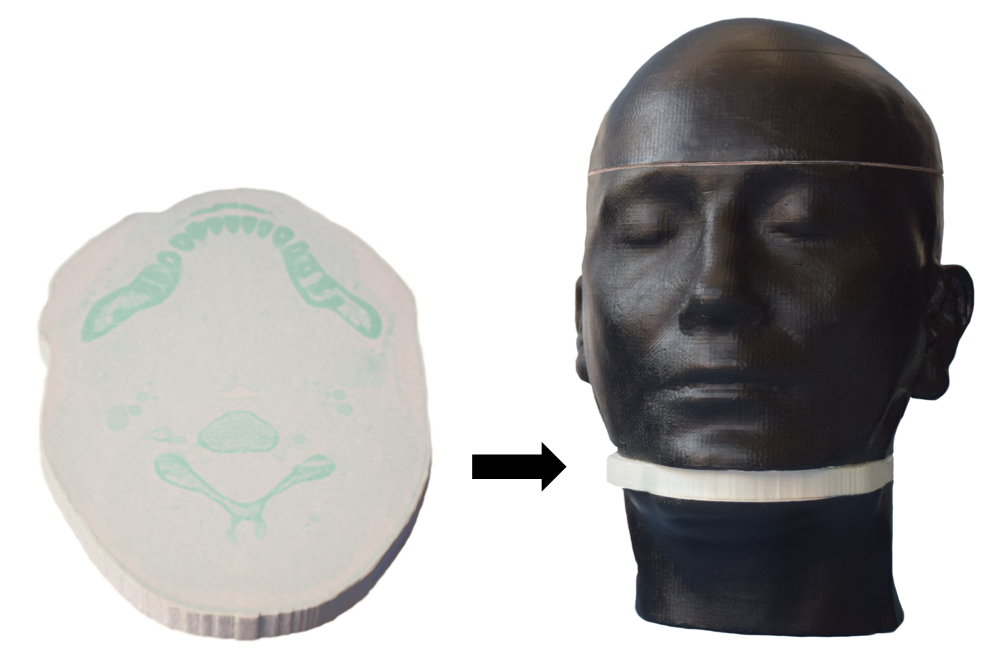


Suppl. fig. 2: CT images acquired with all 36 protocols. Top: Image reconstruction with FBP. Bottom: Image reconstruction with AIDR 3D. Drawings indicate the lesion position. Images are displayed with window level/window width 40/350 at 120 kVp and 80/350 at 100 kVp.


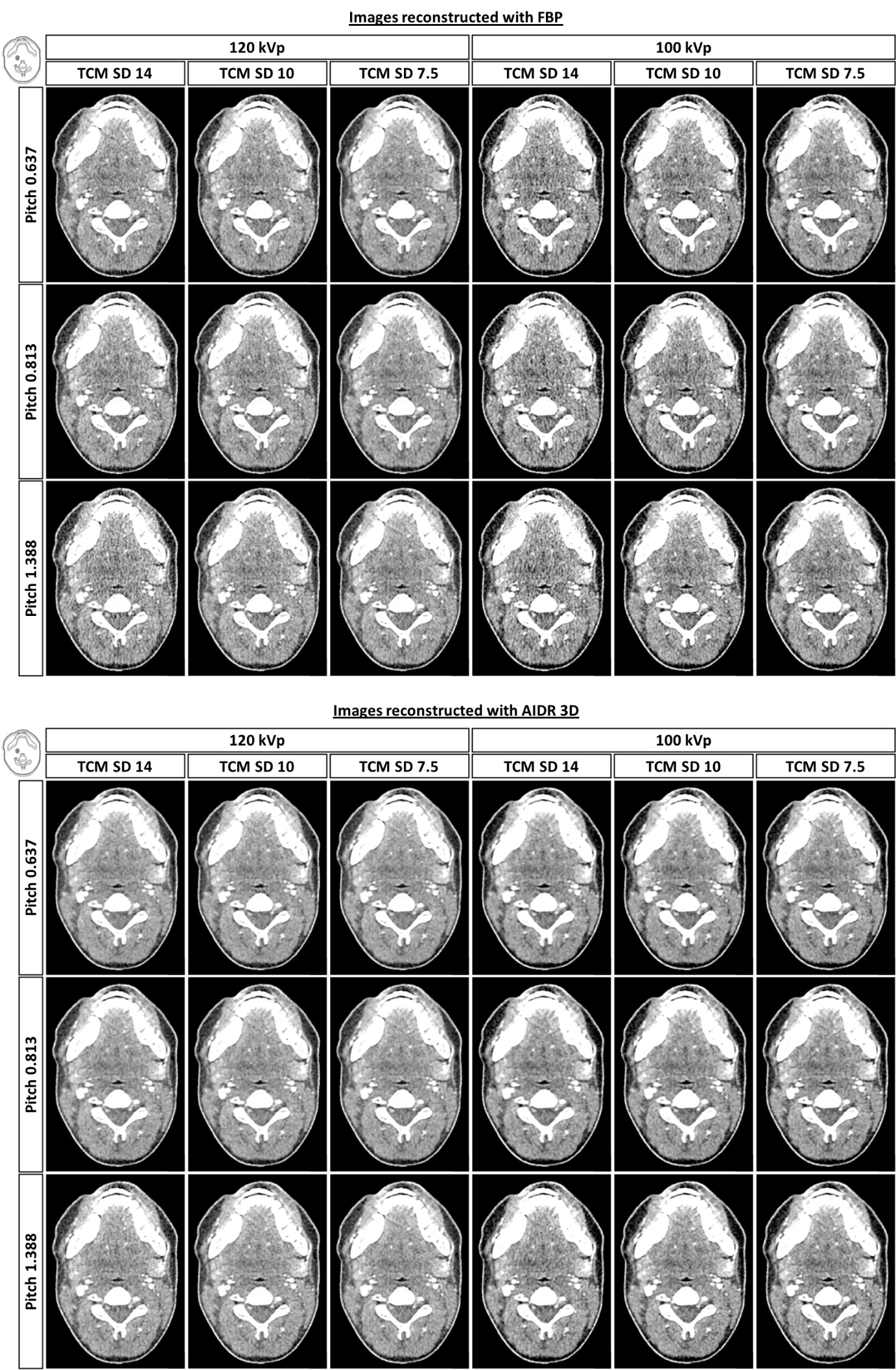

Supplement: Supplementary file 1 — (DOCX 1.32 mb) [file 330_2020_7374_MOESM1_ESM.docx]
